# Papain Ameliorates Lipid Accumulation and Inflammation in High-Fat Diet-Induced Obesity Mice and 3T3-L1 Adipocytes via AMPK Activation

**DOI:** 10.3390/ijms22189885

**Published:** 2021-09-14

**Authors:** Yun-Mi Kang, Hyun-Ae Kang, Divina C. Cominguez, Su-Hyun Kim, Hyo-Jin An

**Affiliations:** 1 Department of Pharmacology, College of Korean Medicine, Sangji University, Wonju 26339, Gangwon-do, Korea; yunmi6115@naver.com (Y.-M.K.); Kha5414@hanmail.net (H.-A.K.); divina_0406@yahoo.com (D.C.C.); 2 Department of Obstetrics and Gynecology, College of Korean Medicine, Sangji University, Wonju 26339, Gangwon-do, Korea; tngusl87@sangji.ac.kr

**Keywords:** papain, *Carica papaya*, obesity, inflammation, high-fat diet, 3T3-L1 preadipocytes, adipogenesis, AMP-activated protein kinase

## Abstract

Papain is a proteolytic enzyme present in the leaves, fruits, roots, and latex of the *Carica papaya* (papaya) plant. Although it exhibits a wide range of activities, there are no reports on the anti-obesity effects of papain. This study examined the anti-obesity effect and obesity-involved anti-inflammatory mechanism of papain in in vivo and in vitro models using high-fat diet (HFD)-induced obese mice and 3T3-L1 preadipocytes. Oral administration of papain reduced HFD-induced weight of the body, liver, and adipose tissues of mice. Papain also reduced hepatic lipid accumulation and adipocyte size. Moreover, serum total cholesterol and triglyceride levels were markedly reduced in papain-treated mice. In addition, papain inhibited the differentiation of preadipocytes and oil accumulation in 3T3-L1 preadipocytes and rat primary preadipocytes. Mechanistically, papain significantly downregulated the protein levels of key adipogenesis regulators and reversed the expression of pro-inflammatory cytokines and adipokines in HFD-induced obese mice and 3T3-L1 preadipocytes. Papain also markedly enhanced activation of the AMP-activated protein kinase pathway in both models. Collectively, these results suggest that papain exerts anti-obesity effects in HFD-induced mice and 3T3-L1 preadipocytes by regulating levels of adipogenic factors involved in lipid metabolism and inflammation; thus, it could be useful in the prevention and treatment of obesity.

## 1. Introduction

Obesity is a major risk factor that contributes toward a number of chronic diseases, including diabetes, hypertension, hyperlipidemia, cardiovascular diseases, and cancer with its prevalence increasing to epidemic proportions in recent decades [1]. Most drugs for the treatment of obesity have been withdrawn from the market due to their adverse effects; hence, the current conventional therapeutic options for the treatment of obese people is not sufficient [2]. Research has shown that diverse natural sources have the potential to be developed as natural anti-obesity agents for their biological actions and the presence of active phytochemical constituents [3].

Adipogenesis is defined as the process by which preadipocytes differentiate into adipocytes. This process can be explained in two steps: an increase in the number of preadipocytes and the differentiation of preadipocytes into mature adipocytes [4]. Differentiation of preadipocytes into adipocytes is regulated by a complex network of transcription factors responsible for the expression of key proteins that induce mature adipocyte formation. The process of adipogenesis also involves changes in cell morphology, induction of insulin sensitivity, and changes in the secretory capacity of cells at different stages of differentiation. In mammalian cells, CCAAT/enhancer binding protein α (C/EBPα), peroxisome proliferator-activated receptor γ (PPARγ), and sterol regulatory element-binding protein (SREBP)-1 are the main regulators of adipogenesis [5]. C/EBPs play an important role in activating PPARγ expression during the early stages of differentiation [6], PPARγ promotes lipogenic gene expression, and SREBP-1 induces fatty acid synthesis, such as acetyl-CoA carboxylase and fatty acid synthase [7]. Meanwhile, obesity is an underlying condition of inflammatory and metabolic diseases. As a strong risk factor for many diseases, obesity predisposes the body to a pro-inflammatory state via increased levels of inflammatory mediators such as tumor necrosis factor (TNF)-α, interleukin (IL)-6, leptin, and reduced levels of adiponectin secreted by adipose tissue. This family of cytokines and hormones are collectively referred to as adipokines. These adipokines regulate the proliferation and apoptosis of adipocytes by promoting lipolysis, inhibiting lipid synthesis, and decreasing blood lipids through autocrine and paracrine mechanisms [8]. AMP-activated protein kinase (AMPK) is an important energy regulator for energy metabolism in multiple tissues. It has been reported to inhibit the synthesis of fatty acids, cholesterol, isoprenoids, and hepatic gluconeogenesis while increasing muscle glucose transport, fatty acid oxidation, and caloric intake [9]. In adipose tissue, AMPK is activated in response to a variety of extracellular stimuli. Accordingly, the key role of AMPK in reprograming the metabolism has been heavily pursued as a potential therapeutic avenue in the treatment of several metabolic diseases such as diabetes, obesity, cancer, and inflammation [10]. Thus, AMPK is a molecular target of drugs used in the treatment of metabolic diseases, including obesity.

*Carica papaya*, belonging to the family Caricaceae, is a useful tropical plant eaten as a fresh fruit and in drinks, jams, and candies. In particular, its latex contains enzymes that are used in food, leather, and pharmaceutical industries. Papain is a cysteine protease isolated and characterized from *C. papaya* and is classified as a proteolytic enzyme that requires a free sulfhydryl group for activity [11]. It has been extensively studied for diverse applications in numerous fields such as in the food industry, meat tenderizing, drug design, and pharmaceutical preparations. As a popular folk remedy, papain was used to reduce pain, inflammation, infection, swelling, diarrhea, and allergies, in addition to improving digestion. It is also known for its wound healing properties [12], antibacterial activity [13], and exhibits inhibitory effects on platelet activation [14], monocyte-platelet aggregate formation [15], strongyloidiasis [16], atherosclerosis [17] and peritoneal adhesion [18]. However, its effect on obesity has not been studied. Interestingly, it has been reported that aqueous fruit extract of *C. papaya* has anti-obesity effects in high-fat diet fed obese rats [19] and the aqueous extract of its leaves exerts anti-hyperglycemic and hypolipidemic activities in an alloxan-induced diabetic rat model [20]. It is considered that *C. papaya* may contribute to the prevention and treatment of obesity and its associated metabolic disorders [21]. Moreover, *C. papaya* is used in the treatment of inflammatory conditions and has shown anti-inflammatory effects [22,23]. Therefore, we speculated that papain could be effective in reducing obesity-associated inflammation. Studies have additionally shown papain to be associated with the prevention of peritoneal adhesion formation, which is caused by an inflammatory response [24,25,26], further supporting our hypothesis. In this study, we conducted in vivo and in vitro experiments to explore the effect of papain on obesity, and to elucidate its mechanisms of action in a high-fat diet (HFD)-induced obese mouse model, 3T3-L1 adipocytes and rat primary adipocytes. The results demonstrated that papain inhibits adipogenesis and reduces obesity and obesity-induced inflammation via inhibition of adipogenesis factors and pro-inflammatory cytokines through AMPK activation.

## 2. Results

### 2.1. Papain Decreased Fat Accumulation, Body Weight and Serum Lipid Content in HFD-Induced Obese Mice

**Figure 1:**
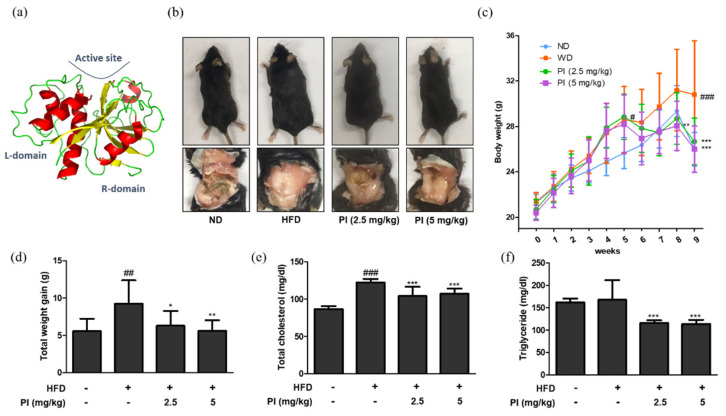
Effects of papain on macroscopic changes and serum profiles in HFD-induced obese mice. (**a**) Three–dimensional structure of papain. (**b**) Representative macroscopic pictures of HFD–induced obese mice from different groups; C57BL/6 mice (upper panel) and abdomen of mice (lower panel). (**c**) Body weight and (**d**) weight gain were measured once a week for a duration of 9 weeks. A comparison was made between the total weight gain obtained at the last week compared to the first week. (**e**) Serum levels of total cholesterol and (**f**) triglyceride in HFD-induced obese mice. The values are given as the mean ± SD (*n* = 6). ^#^ *p* < 0.05, ^##^ *p* < 0.01 and ^###^ *p* < 0.001 vs. ND group; * *p* < 0.05, ** *p* < 0.01 and *** *p* < 0.001 vs. HFD group.

The three-dimensional structure of papain is shown in Figure 1a. To investigate the effects of papain on obesity, mice were fed an HFD for 9 weeks to develop obesity, followed by administering papain (2.5, or 5 mg/kg) for the last 4 weeks. As shown in Figure 1b, the feeding of an HFD for 9 weeks significantly increased abdominal fat accumulation in the HFD mice compared to the normal diet (ND) group. In contrast, the mice fed with an HFD, but treatment with papain showed markedly decreased fat accumulation at 9 weeks compared to those in the HFD group. Weekly body weight results also showed that the weight of HFD-fed mice was consistently higher than that of the ND group. However, the administration of papain significantly decreased the body weight compared to that in the HFD group from the 8th week (Figure 1c). The total weight gain of mice in the papain treated group was significantly lower than that in the HFD group at the end of the feeding period (Figure 1d). Obesity is accompanied by abnormal triglyceride and cholesterol levels, and lipid metabolism. As shown in Figure 1e,f, HFD feeding significantly increased the serum level of total cholesterol but not triglycerides compared to the ND group. However, the serum levels were significantly reduced in the papain treatment compared to the HFD group.

### 2.2. Papain Decreased the Liver Weight and Liver Lipid Accumulation in HFD-Induced Obese Mice

**Figure 2:**
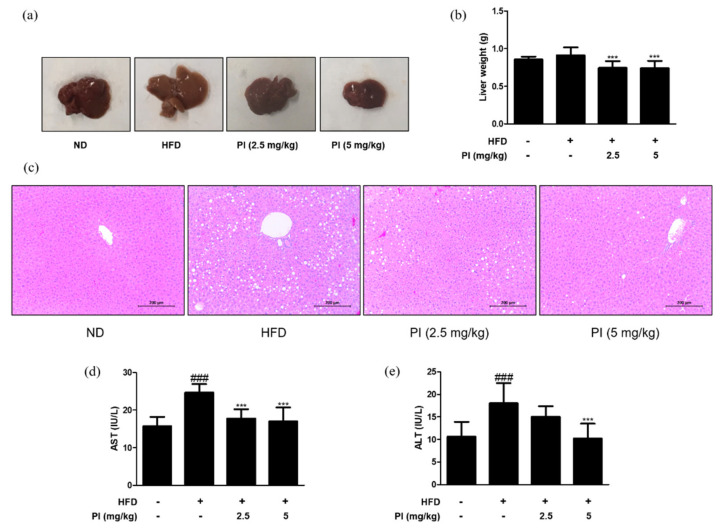
Effects of papain on liver steatosis in HFD–induced obese mice. (**a**) Macroscopic analysis of mouse liver tissue from different groups in HFD–induced obese mice. (**b**) Liver weight in HFD–induced mice. (**c**) Representative histological images of the liver tissue were assessed using H&E staining (original magnification; 200×). The liver tissue from representative mice in each group was fixed, embedded in paraffin, and stained with H&E. (**d**) Serum levels of AST and (**e**) ALT in HFD–induced obese mice. The values are given as the mean ± SD (*n* = 6). ^###^ *p* < 0.001 vs. ND group; *** *p* < 0.001 vs. HFD group.

To check the effect of papain on liver lipid accumulation, the liver color was observed, and the weight of liver tissue was measured in each group. The liver color of the HFD group appeared light yellow and that of the ND group was dark red, while the liver color of the HFD group treated with papain changed to dark red (Figure 2a). Although there was no difference in the liver weight between the ND and HFD groups at 9 weeks, HFD mice treated with papain showed a significant decrease in the liver weight compared to the HFD-fed mice (Figure 2b). Histopathological analysis of the liver tissue showed a significantly larger number of microscopically identifiable lipid droplets and higher lipid accumulation in the HFD group than in the ND group. Meanwhile, the administration of papain resulted in a notable decrease in these lipid droplets (Figure 2c). Additionally, the administration of papain to mice had no significant toxic effects on AST and ALT, which are sensitive indicators of liver damage, suggesting that papain induced a reduction in body weight and lipid accumulation without any toxicity in HFD-induced obese mice (Figure 2d,e).

### 2.3. Papain Regulated the Expression of Adipogenic and Inflammatory Markers in Liver of HFD-Induced Obese Mice

**Figure 3:**
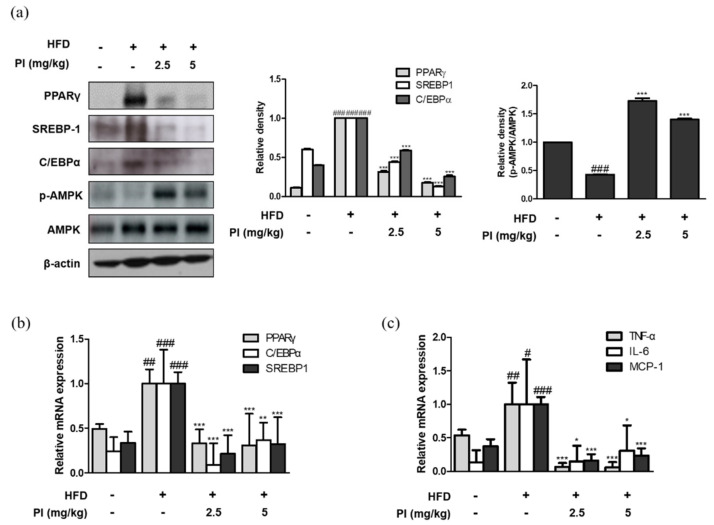
Effects of papain on adipogenic factors and inflammatory markers in liver of HFD–induced obese mice. (**a**) Protein expressions of PPARγ, SREBP–1, C/EBPα, p–AMPK and AMPK were determined by western blot analysis with specific antibodies. β-actin was used as internal controls. (**b**) The mRNA levels of PPARγ, SREBP–1, and C/EBPα were determined by qRT–PCR. (**c**) The mRNA levels of TNF–α, IL–6, and MCP–1 were determined by qRT–PCR. Densitometric analysis was performed using Bio-Rad Quantity One^®^ Software. The data shown represent mean ± SD of three independent experiments. ^#^ *p* < 0.05, ^##^ *p* < 0.01, ^###^ *p* < 0.001 vs. ND group; * *p* < 0.05, ** *p* < 0.01 and *** *p* < 0.001 vs. HFD group.

To investigate the molecular basis for the anti-obesity effect of papain, the mRNA and protein expression of adipogenic transcription factors were measured. As shown in Figure 3a, the protein expressions of PPARγ, C/EBPα, and SREBP-1 were increased in liver of HFD-fed mice. However, administration of papain significantly decreased the protein expression levels of these markers in the livers of HFD mice. The mRNA expressions of PPARγ, C/EBPα, and SREBP-1 were also downregulated by papain treatment in HFD-fed mice (Figure 3b). It is well known that the AMPK signaling pathway regulates the expressions of adipogenic factors [27]. Therefore, we examined whether papain influences AMPK activation. The protein expression of phosphorylated AMPK was decreased in the HFD group compared to that in the ND group, whereas it was significantly increased by papain treatment (Figure 3a). Moreover, the mRNA expression levels of the pro-inflammatory cytokines TNF-α, IL-6, and MCP-1 were significantly increased in the HFD group compared to levels in the ND group, but the expression levels in HFD mice treated with papain were significantly decreased compared with those in the HFD group (Figure 3c).

### 2.4. Papain Reduced the Adipose Fat Weight and Inhibited the Adipocyte Hypertrophy in HFD-Induced Obese Mice

**Figure 4:**
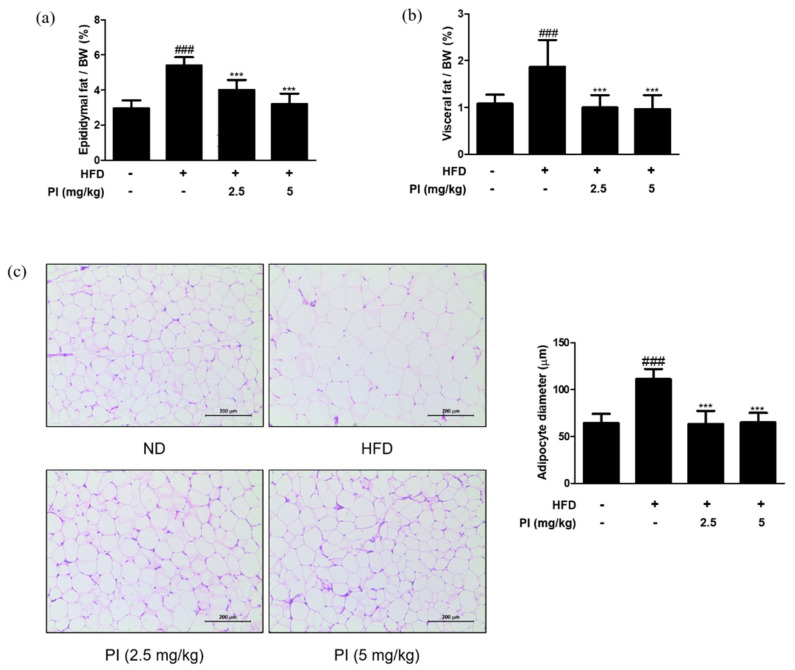
Effects of papain on adipose tissue weight and adipocyte hypertrophy in HFD–induced obese mice. (**a**) Relative epididymal adipose tissue weight ratio and (**b**) relative visceral adipose tissue weight ratio measured after 9 weeks of diet treatments in mice. (**c**) Representative histological images of the epididymal adipose tissue were assessed using H&E staining (original magnification; 200×). The epididymal white adipose tissue from representative mice in each group was fixed, embedded in paraffin, and stained with H&E. Adipocyte diameter was quantified under microscope quantified from representative sections. The values are given as the mean ± SD (*n* = 6). The values are given as the mean ± SD (*n* = 6). ^###^ *p* < 0.001 vs. ND group; *** *p* < 0.001 vs. HFD group.

White adipose tissue is the major site of lipid storage, and its main depots in rodents include epididymal, visceral, and mesenteric pads [28]. Thus, we measured the ratios of epididymal fat weight to body weight and visceral fat weight to body weight. As shown in Figure 4a,b, ratios of both the epididymal and visceral fat weight to body weight in the HFD group were increased by approximately two times compared to the ND group. However, treatment with papain significantly reduced the epididymal and visceral fat weight to body weight ratios when compared to the HFD group. Histological analysis showed larger adipocytes in epididymal adipose tissue of the HFD group (111.0 ± 11.06 μm) than that in the ND group (64.1 ± 9.95 μm). Treatment with papain remarkably decreased adipocyte enlargement to a level similar to that in the ND group (Figure 4c). These results demonstrate that papain administration effectively prevents lipid accumulation and adipocyte hypertrophy.

### 2.5. Papain Regulated Adipokines and Macrophage Infiltrations in Adipose Tissue of HFD-Induced Obese Mice

**Figure 5:**
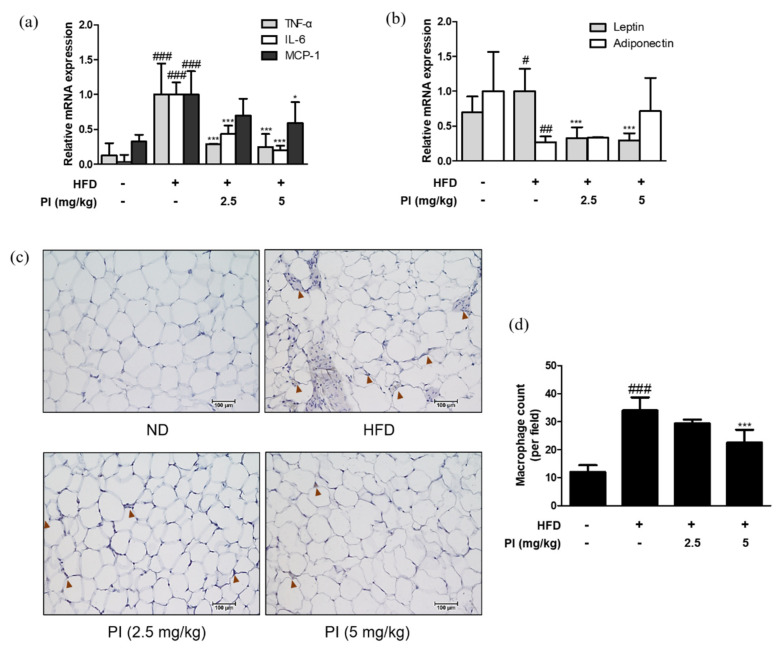
Effects of papain on adipokines and F4/80 expressions in HFD–induced obese mice. (A) The mRNA levels of (**a**) TNF–α, IL–6, MCP–1, (**b**) leptin and adiponectin were determined by qRT–PCR. (**c**) Epididymal adipose tissue sections were stained with immunohistochemical staining (*n* = 6). F4/80 in the mouse epididymal adipose tissue were detected using specific antibodies. Brown arrows indicated stained. Bars indicate 100 μm. (**d**) Quantification of stained macrophages. ^#^ *p* < 0.05, ^##^ *p* < 0.01, ^###^ *p* < 0.001 vs. ND group; * *p* < 0.05, *** *p* < 0.001 vs. HFD group.

To confirm the presence of inflammation in the adipose tissue of HFD-fed mice, we assessed the expression levels of adipokines. HFD resulted in a significant increase in the mRNA expression levels of TNF-α, IL-6, MCP-1, and leptin. However, these levels were downregulated by papain treatment (Figure 5a). Moreover, we observed the decreased mRNA expression of adiponectin in HFD mice compared to that in the ND group; while it was upregulated by papain treatment, the difference was not significant (Figure 5b). The F4/80 expression was significantly increased with higher levels of macrophage infiltration surrounding adipocytes in the adipose tissue of HFD-fed mice compared to the ND group. However, this infiltration was decreased by papain treatment (Figure 5c,d). These results suggest that the weight loss of adipose tissue in the papain treatment group could be attributed to the inhibition of lipid accumulation and the hypertrophy of adipocytes due to the reduction of adipocyte differentiation and the regulation of inflammatory responses.

### 2.6. Papain Decreased Adipocyte Differentiation in 3T3-L1 Preadipocytes and Primary Preadipocytes

**Figure 6:**
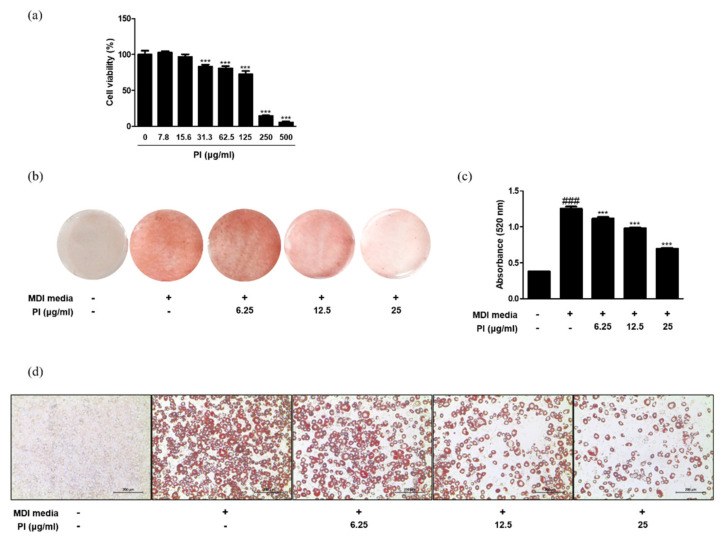
Effects of papain on adipocyte differentiation in MDI–treated 3T3–L1 preadipocytes. (**a**) Cell viability of papain in 3T3–L1 preadipocytes. *** *p* < 0.001 vs. non-treated group. (**b**) Oil red O staining 8 days after induction. (**c**) Quantitative analysis of Oil Red O staining. Oil Red O stained cells were extracted with isopropyl alcohol and the absorbance at 520 nm was measured. (**d**) Macroscopic shots of oil red O staining are shown. The values are given as the mean ± SD ^###^ *p* < 0.001 vs. non-treated group; *** *p* < 0.001 vs. MDI-treated group.

To examine the cytotoxicity of papain in 3T3-L1 preadipocytes, the cells were treated with a range of concentrations (0, 7.8, 15.6, 31.3, 62.5, 125, 250, and 500 μg/mL) of papain, and cell viability was determined using the MTT assay. The viability of cells treated with papain markedly decreased from 31.3 μg/mL in a concentration-dependent manner (Figure 6a). To focus on the non-cytotoxic range of papain, the concentrations of 6.25, 12.5, and 25 μg/mL were used in the following experiments. To bring about in vitro adipocyte differentiation, 3T3-L1 preadipocytes were induced by the addition of MDI medium. The number of Oil Red O-stained adipocytes increased by treatment with MDI medium, but subsequently reduced upon papain treatment (Figure 6b–d). To verify the effect of papain on adipocyte differentiation in a fairly representative in vivo tissue environment, we isolated primary preadipocytes from Sprague Dawley rat epididymal fat tissue and stained the cells with Oil Red O. The results showed that papain (25 μg/mL) treatment significantly inhibited cell differentiation and oil accumulation induced by MDI media in preadipocytes from rat to a level similar to that of the normal group (Supplementary Figure S1a–c).

### 2.7. Papain Inhibited the Expression of Adipogenic Markers in MDI-Treated 3T3-L1 Preadipocytes

**Figure 7:**
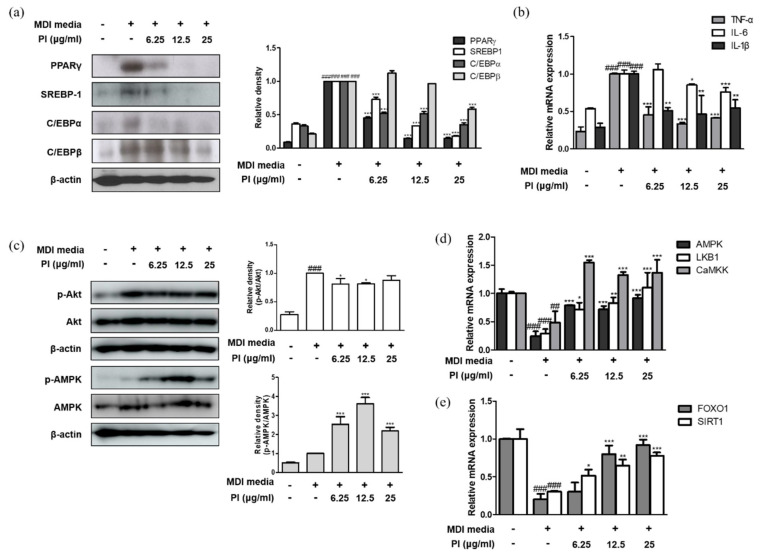
Effects of papain on inflammatory and adipogenic factors in MDI–treated 3T3–L1 preadipocytes. (**a**) Protein expressions of PPARγ, SREBP–1, C/EBPα and C/EBP β were determined by western blot analysis with specific antibodies. β–actin was used as internal controls. (**b**) The mRNA levels of TNF–α, IL–6, and IL–1β were determined by qRT–PCR. (**c**) Protein expressions of p–AMPK and AMPK were determined by western blot analysis with specific antibodies. β–actin was used as internal controls. The mRNA levels of (**d**) AMPK, LKB1, CaMMK, (**e**) FoxO1, and SIRT1 were determined by qRT–PCR. Densitometric analysis was performed using Bio-Rad Quantity One^®^ Software. The data shown represent mean ± SD of three independent experiments. ^##^ *p* < 0.01, ^###^ *p* < 0.001 vs. non-treated group; * *p* < 0.05, ** *p* < 0.01, *** *p* < 0.001 vs. MDI-treated group.

Next, we investigated the effect of papain on adipogenesis in 3T3-L1 preadipocytes. As shown in Figure 7a, papain significantly decreased the protein expression levels of the key adipogenic transcription factors PPARγ, SREBP-1, C/EBPα, and C/EBPβ in MDI-treated cells. Furthermore, the mRNA expression levels of TNF-α, IL-6, and IL-1β were enhanced by inducing adipocyte differentiation; however, papain treatment significantly inhibited the expression of pro-inflammatory cytokines in MDI-treated cells (Figure 7b). These results suggest that papain treatment prevents adipocyte differentiation by downregulating the expression of adipogenesis-inducing markers in 3T3-L1 preadipocytes.

### 2.8. Papain Regulated the Expression of AMPK/SIRT1-FoxO1 in MDI-Treated 3T3-L1 Preadipocytes

We investigated whether the inhibition of adipocyte differentiation by papain was mediated by AMPK activation in 3T3-L1 preadipocytes. Consistent with the phosphorylation of AMPK in liver of HFD mice treated with papain, the phosphorylation of AMPK was significantly increased by papain in 3T3-L1 preadipocytes too; while papain (6.25 and 12.5 μg/mL) suppressed the MDI-induced phosphorylation of Akt, which is involved in the adipocyte differentiation and fatty acid synthesis in 3T3-L1 preadipocytes (Figure 7c). Papain treatment significantly upregulated the mRNA expression levels of AMPK, liver kinase B1 (LKB1), and calcium/calmodulin-dependent protein kinase (CaMKK), which are well-known upstream regulators of AMPK (Figure 7d). As the transcription factor forkhead box O1 (FoxO1) plays an important role in white adipocyte differentiation and metabolic homeostasis [29]—and Sirtuin (SIRT)1, like AMPK, is involved in cellular processes such as energy and lipid metabolism and mitochondrial biogenesis, and controls adipokines in the adipose tissue [30]—we measured the effects of papain on these markers. Adipogenesis induced by the MDI medium attenuated the mRNA expressions of FoxO1 and SIRT1 compared to non-treated cells, and papain treatment significantly upregulated the levels of FoxO1 and SIRT1 in MDI-treated 3T3-L1 preadipocytes (Figure 7e).

## 3. Discussion

In this study, we observed the anti-obesity and anti-inflammatory effects of papain in HFD-fed mice and MDI-treated 3T3-L1 preadipocytes. Treatment with papain ameliorated obesity by reducing body, liver, adipose fat tissue weight, and serum lipid profiles in HFD-fed mice (Figure 1, Figure 2 and Figure 4). With adipose tissue, the liver is a key metabolic organ which plays a central role in lipid metabolism such as lipid distribution and energy metabolism [31]. In the condition of over-nutrition and obesity, the altered lipid metabolism leads to the accumulation of triglycerides in the liver through adipogenesis and lipogenesis, and to a clinical condition known as non-alcoholic fatty liver disease (NAFLD) [32]. In this study, papain treatment noticeably suppressed the increased expression levels of adipogenesis-related transcription factors, including PPARγ, C/EBPα, and SREBP-1, in the liver induced by HFD feeding and MDI-treated 3T3-L1 preadipocytes (Figure 3 and Figure 7). PI3K/Akt signaling is important in the upregulation of these transcription factors and adipogenesis [33]. The Akt activation was also inhibited by papain in MDI-treated 3T3-L1 preadipocytes in this study (Figure 7c). Our results indicated that papain suppressed adipogenesis through the PPARγ, C/EBPα, and SREBP-1 downregulation via inhibition of Akt activation. Upon treatment with papain, furthermore, adipocyte differentiation was evidently suppressed, not only in MDI-stimulated 3T3-L1 preadipocytes (Figure 6) but also in MDI-treated primary preadipocytes derived from rat adipose tissue (Supplementary Figure S1). These results suggest that the weight loss of adipose tissue in the papain treatment group could be attributed to lipids not being utilized for the hypertrophy of adipocytes and the formation and accumulation of fat in the body due to the downregulation of adipogenic factors, all of which, in turn, result in the reduction of adipocyte differentiation. The liver and adipose tissue interplay with each other to maintain energy homoeostasis by the way of several adipokines, and hepatokines, such as IL-6 and acylcarnitine [34]. Papain may exert its beneficial effect through synergy with the metabolic system, suggesting a potential treatment option for NAFLD as well as obesity.

An additional feature of the inflammatory state of obesity is the increased infiltration of immune cells into the metabolic tissues [8]. Then, we focused on the adipose tissue as an active endocrine organ, which contribute to chronic low-grade inflammation that is seen in metabolic diseases such as obesity [35]. Our results showed that papain suppressed levels of pro-inflammatory cytokines, adipokines, and F4/80-expressing macrophages (Figure 3c and Figure 5). As shown in our results, the macrophage population is increased in the adipose tissue of HFD-fed mice compared with mice fed a normal diet, and these cells contribute to the increased cytokine expression [36]. Some findings suggest that alterations in the production of inflammatory biomolecules precede the increased immune cell infiltration and the induction of a macrophage phenotype switch in visceral adipose tissue. Furthermore, serum leptin and resistin have specific roles in the regulation of adipose tissue macrophages in patients with modest obesity or early metabolic dysfunction [37]. Therefore, the decrease in pro-inflammatory cytokines in the obesity milieu implies that papain suppresses obesity conditions that interact with inflammatory responses. Our observations are supported by previous studies showing that increased adipose tissue-derived cytokine levels suppress the synthesis and secretion of adiponectin, which exerts anti-inflammatory actions [38].

The activation of AMPK has been demonstrated in response to a variety of extracellular stimuli in adipose tissue; thus, it is a molecular target of drugs used for the treatment of metabolic diseases, including obesity. In this study, a significant increase in AMPK activation by papain was observed in both HFD-fed mice and MDI-stimulated 3T3-L1 preadipocytes (Figure 3a and Figure 7c). LKB1 is a kinase which acts upstream of AMPK and plays an important regulatory role in glucose metabolism in the muscle, liver, and other tissues. In certain cell types, CaMKKβ has been shown to activate AMPK in response to increases in intracellular Ca^2+^ levels [39]. These upstream markers of AMPK were increased by papain in MDI-stimulated 3T3-L1 preadipocytes indicating that papain appears to decrease differentiation and inflammation via the activation of the AMPK signaling pathway. On the other hand, obesity is a triggering risk factor for type 2 diabetes associated with insulin resistance. Increased adipose tissue mass in obese individuals is a major cause of the ectopic alteration of insulin sensitivity [40]. It has been reported that the activation of AMPK is an attractive target to improves insulin sensitivity and glucose homeostasis [41]. Upon activation, AMPK enhances glucose transport through GLUT4 regulation in insulin-resistant skeletal muscle [42], and it involves lowering the membrane cholesterol [43]. Our result showed that papain enhanced the expression of AMPK in vivo and in vitro; these findings suggest implications in the insulin sensitivity and provide insights into whether the use of papain could be expanded to metabolic syndrome. In line with this assumption, it is reported that the fermented papaya preparation induces a protective effect on platelets from patients with type 2 diabetes, by opposing oxidative damage associated with diabetes and its complications [44], supporting the idea that papain may have potential benefits in the management of insulin resistance mediated by an obese state. However, the effect of papain needs to be verified using the established insulin resistance model.

We have noted that AMPK negatively regulates lipid-induced inflammation, which acts through SIRT1, thereby contributing to the protection against obesity, inflammation, and insulin resistance [45], and the activation of both AMPK and SIRT1 may contribute to its anti-inflammatory effects [46]. Moreover, it has been suggested that the association between deacetylation of LKB1 modulated by SIRT1 and AMPK activation [47]. Meanwhile, AMPK increases intracellular NAD^+^ levels, causing SIRT1 to act as a metabolic sensor to regulate responses [48]. Therefore, targeting AMPK and SIRT1 in adipose tissue may be desirable for normalizing adipose dysfunction and inflammation. It is worth studying the SIRT1-involved signaling pathway and the interaction between AMPK in obesity and inflammation. Further studies are needed to clarify the actions and linking mechanisms of papain on an obesity and inflammation-combined status.

While it is widely accepted that *C. papaya* can help digestive disorders and weight loss by people in the tropics, it is still unclarified the role of papain in beneficial effects; thus, in this study, mice were orally administrated with papain, and the results showed the positive effect against obesity and inflammation. For several decades, *C. papaya* has been used for the treatment of helminth infections; and the oral administration of plant cysteine proteinases has known to be able to damage to the cuticles of parasitic nematodes [49]. It reported that the optimum pH for activity of papain is in the range of 3.0–9.0, and the activity and stability are varied according to environmental factors such as temperature, humidity, metal ions, and the type of substrate being hydrolyzed. Interestingly, papain might be only partially folded by the stomach acidity forming intermediate states known as molten globule [50]. Therefore, it is implied that some benefits of papain such as digestibility would be helpful for management of metabolic disease including obesity and inflammation, although it is required to consider for better activity of papain to be more effective in the gut.

In conclusion, the current study revealed that papain treatment could inhibit obesity progression by regulating lipid accumulation and lowering blood lipid levels through the downregulation of adipogenic factors including PPARγ, C/EBPα, and SREBP-1 in HFD-induced obese mice and 3T3-L1 preadipocytes. Moreover, papain decreased inflammation associated with obesity by inhibiting pro-inflammatory cytokines, leptin, and increased adiponectin through AMPK activation. The results of this study indicate that papain, a natural product, has the potential to be developed as an agent for the treatment of obesity and other obesity-associated inflammatory diseases.

## 4. Materials and Methods

### 4.1. Chemicals and Reagents

Papain (from papaya latex, P4762), isobutyl methyl xanthine (IBMX), dexamethasone (DEX), insulin, Oil red O, and all other chemicals were purchased from Sigma-Aldrich (St. Louis, MO, USA). HFD (45%) was purchased from Research Diets (New Brunswick, NJ, USA). Dulbecco’s modified Eagle’s medium (DMEM), DMEM-F12, bovine serum (BS), heat-inactivated fetal bovine serum (FBS), and penicillinstreptomycin were purchased from Life Technologies, Inc. (Grand Island, NY, USA). Primary antibodies against SREBP-1, PPARγ, C/EBPα, C/EBPβ, and β-actin antibodies were purchased from Santa Cruz Biotechnology, Inc. (Santa Cruz, CA, USA). Primary antibodies against p-AMPK and AMPK were purchased from Cell Signaling Technology, Inc. (Danvers, MA, USA). Horseradish peroxidase-conjugated secondary antibodies were obtained from Jackson Immuno Research Laboratories, Inc. (West Grove, PA, USA). SYBRTM Green PCR Master Mix was purchased from Applied Biosystems (Foster City, CA, USA). Oligonucleotide primers for PPAR, SREBP1, C/EBP, and GAPDH were purchased from Bioneer (Daejeon, Chungbuk, Republic of Korea).

### 4.2. Animal Experiments

Twenty-four 4-week-old C57BL/6J mice (male, 20–25 g) were purchased from Daehan Biolink Co. (Daejeon, South Korea) and maintained under constant conditions (temperature, 22 ± 3 °C; humidity, 40%–50%; light/dark cycle 12 h/12 h). Mice were adapted to the feeding conditions for 1 week and then given free access to food and tap water for 9 weeks. Mice were randomly separated into groups of 6 each: ND group, HFD (45% fat diet, D12451) group, and HFD treated with papain (2.5 or 5 mg/kg). With the exception of the ND group, all mice were fed an HFD. Papain was dissolved in PBS and orally administered on a daily basis for the last 4 weeks along with HFD in the 2.5 or 5 mg/kg papain groups. Body weights were recorded weekly. On the last day of 9th week, the animals were fasted overnight. Blood samples were collected via cardiac puncture. The liver and epididymis fat tissues were excised, rinsed, and directly stored at −80 °C until analysis. All experiments were approved by the Ethical Committee on Animal Care and the Use of Laboratory Animals at Sangji University (Registration no. 2018-22).

### 4.3. Histological Analysis

Liver and epididymis fat tissues were fixed with 4% formaldehyde solution (10% formalin) and embedded in paraffin. For histological analysis, sections of the samples (8 μm thickness) were stained with hematoxylin and eosin (H&E). The stained slides were observed using a Leica DM IL LED microscope (Leica, Wetzlar, Germany).

### 4.4. Analysis of Serum Lipid Profile

Blood samples were collected and centrifuged at 1700× *g* for 15 min at room temperature to obtain serum samples. Unused samples were immediately frozen at −70 °C for subsequent measurements. Serum levels of total cholesterol, AST, and ALT were determined by enzymatic methods using commercial kits (AM202, AM103-K, AM102, ASAN PHARMACEUTICAL, Seoul, Korea).

### 4.5. Reverse Transcription-Quantitative Polymerase Chain Reaction (RT-qPCR) Analysis

Total RNA was isolated from the liver tissues, adipose tissues, or cells using an Easy Blue kit (Intron Biotechnology, Inc., Seoul, Korea) according to the manufacturer’s protocol. Total RNA was quantified using an Epoch micro-volume spectrophotometer system (BioTek Instruments, Inc., Winooski, VT, USA). cDNA was obtained using isolated total RNA (2 μg), d(T)16 primer, and Avian Myeloblastosis Virus reverse transcriptase with genomic DNA remover. Relative gene expressions were quantified using RT-qPCR analysis (Real Time PCR System 7500; Applied Biosystems; Thermo Fisher Scientific, Inc., Waltham, MA, USA) with SYBR Premix Ex Taq. Fold changes in gene expression were calculated using the comparative quantification cycle method. The Cq values of target genes SREBP-1, PPARγ, C/EBPα, TNF-α, IL-6, IL-1β, MCP-1, leptin, adiponectin, FOXO1, SIRT1, AMPK, LKB1, and CaMKK were normalized to that of GAPDH using the ABI Gene Express 2.0 program (Applied Biosystems; Thermo Fisher Scientific, Inc., Waltham, MA, USA). The sequences of oligonucleotide primers are described in Table 1.

### 4.6. Western Blot Analysis

Liver tissues and cells were homogenized in PRO-PREP™ protein extraction solution (Intron Biotechnology, Seoul, Republic of Korea) and were incubated for 30 min at 4 °C. Debris was removed by micro-centrifugation at 11,000× *g*, followed by the quick freezing of the supernatants. Protein concentration was determined using the Bio-Rad protein assay reagent according to the manufacturer’s instructions (Bio-Rad, Hercules, CA, USA). Proteins were electroblotted onto a polyvinylidene difluoride (PVDF) membrane following separation on a 10%–12% SDS polyacrylamide gel. The membrane was incubated for 1 h with blocking solution (5% skim milk) at room temperature, followed by overnight incubation with primary antibodies at 4 °C. Blots were washed three times with Tween 20/Tris-buffered saline (T/TBS) and incubated with a horseradish peroxidase-conjugated secondary antibody (dilution, 1:2500) for 2 h at room temperature. The blots were again washed three times with T/TBS, and then developed using an enhanced chemiluminescence reagent (GE Healthcare, Waukesha, WI, USA).

### 4.7. Immunohistochemical (IHC) Analysis

Adipose tissues were fixed in 10% buffered formalin, embedded in paraffin, sectioned into 4 μm thick and IHC staining was performed. After paraffin embedding, sections were cut, deparaffinized with xylene, rehydrated in ethanol, and rehydrated with water. Endogenous peroxidase activity was blocked using 0.6% H_2_O_2_ in 50% MeOH, and the slides were then treated with 0.3% Triton in PBS for permeabilization and blocked with 10% normal goat serum for 1 h, followed by overnight incubation with a specific antibody for F4/80 at 4 °C. The sections were then washed and incubated with horseradish peroxidase-conjugated secondary antibodies for 1 h at room temperature. The activity was visualized using a DAB chromogen and counterstained with H&E. Pathological changes in all stained skin sections were observed using a DM IL LED microscope (Leica, Wetzlar, Germany) and photographed using a DFC295 (Leica, Wetzlar, Germany). Digital images were taken from each slide (two per group) and measured using the Leica Application Suite (Leica, Wetzlar, Germany).

### 4.8. 3T3-L1 Preadipocytes Culture and Cell Viability

3T3-L1 preadipocytes were obtained from the American Type Culture Collection (ATCC, Manassas, VA, USA). 3T3-L1 mouse preadipocytes were cultured in DMEM supplemented with 10% BS, 100 units/mL penicillin, and 100 μg/mL streptomycin at 37 °C in 5% CO_2_. Cell viability was assessed using the MTT assay. Briefly, cells were treated with each of the samples and incubated for 24 h, followed by incubation with the MTT solution (5 mg/mL) for 4 h at 37 °C. After discarding the supernatant, the insoluble formazan product was dissolved in DMSO. Cell viability was measured at 540 nm using a microplate reader (Titertek Multiskan, Huntsville, AL, USA).

### 4.9. Rat Primary Preadipocytes Isolation

The isolation and culture of primary preadipocytes from male SD rat epididymal fat tissue were performed as previously described [51] with some modifications. Briefly, epididymal fat pads from rats were removed under sterile conditions, washed with saline solution, minced, and incubated at 37 °C with collagenase type I (17100017, Gibco, Gaithersburg, MD, USA). The digested tissues were filtered through a sterile 100 μm cell strainer. The isolated primary preadipocytes were resuspended in DMEM-F12 medium containing rosiglitazone before the start of the assays.

### 4.10. Adipocyte Differentiation and Oil Red O Staining

Two days after the cells had reached confluence (day 0), they were stimulated with IBMX, DEX, and insulin (MDI cocktail) induction medium (DMEM medium containing 10% heat-inactivated FBS, 0.5 mM IBMX, 1 μm/mL DEX, and 1 μg/mL insulin) and were treated with various concentrations of papain (6.25, 12.5, and 25 μg/mL). Three days after stimulation with MDI (day 3), the cells were cultured in an insulin medium (DMEM medium containing 10% heat-inactivated FBS and 1 μg/mL insulin) and were treated with various concentrations of papain. Three days later (day 6), the cells were cultured in 10% FBS/DMEM and treated with various concentrations of papain. In the case of rat primary adipocytes, MDI medium (DMEM-F12 medium containing 5% heat-inactivated FBS, 0.5 mM IBMX, 1 μm/mL DEX, and 5 μg/mL insulin) and insulin medium (DMEM-F12 medium containing 5% heat-inactivated FBS and 5 μg/mL insulin) were used. Full differentiation was achieved on day 8. Differentiated cells were fixed in 10% formalin for 60 min. Thereafter, the fixing solution was discarded and the cells were incubated in a staining solution of Oil Red O (Sigma Chemical Co., St. Louis, MO, USA) for 3 h. Next, the cells were destained with 40% isopropanol. Oil red O was eluted by incubating the cells in 1 mL of 100% isopropanol for 10 min. Lipid accumulation in the samples was quantified by measuring the optical densities of the samples using a spectrophotometer at 520 nm.

### 4.11. Statistical Analysis

Data are expressed as the mean ± standard deviation (SD) of triplicate experiments. Statistical significance was determined using ANOVA and Dunnett’s post hoc test, and P-values of less than 0.05 were considered statistically significant.

## Figures and Tables

**Table 1:** List of primer sequences.

Gene	Sequence (Forward)	Sequence (Reverse)
SREBP-1	CATCGCAAACAAGCTGACCT	AGATCCAGGTTTGAGGTGGG
PPARγ	ATCGAGTGCCGAGTCTGTGG	GCAAGGCACTTCTGAAACCG
C/EBPα	TCGGTGCGTCTAAGATGAGG	TCAAGGCACATTTTTGCTCC
TNF-α	ATGAGCACAGAAAGCATGAT	TACAGGCTTGTCACTCGAAT
IL-1β	CCCCAAAAGATGAAGGGCTG	CTGGAAGGTCCACGGGAAAG
IL-6	TTCCATCCAGTTGCCTTCTTG	GGGAGTGGTATCCTCTGTGAAGTC
MCP-1	AGGTCCCTGTCATGCTTCTG	TCTGGACCCATTCCTTCTTG
leptin	CTCCAAGGTTGTCCAGGGTT	AAAACTCCCCACAGAATGGG
adiponectin	CTGGAGGTGGGAGACCAAGT	TGGGCTATGGGTAGTTGCAG
FOXO1	CCGGAGTTTAACCAGTCCAA	TGCTCATAAAGTCGGTGCTG
SIRT1	TGCCATCATGAAGCCAGAGA	AACATCGCAGTCTCCAAGGA
AMPK	GGTGGATTCCCAAAAGTGCT	AAGCAGTGCTGGGTCACAAG
LKB1	AAGGGGACGAGGACAAAGAG	GTACTTGCCGATGAGCTTGG
CaMKK	CCCAGTTGGTCCTCTCTGTT	AGAGATCTGAGTACGCCAGC
GAPDH	CCCACTCTTCCACCTTCGAT	CCACCACCCTGTTGCTGTAG

## Data Availability

The data presented in this study are available on request from the corresponding author.

## Supplementary Materials

The following are available online at https://www.mdpi.com/article/10.3390/ijms22189885/s1.
