# Supplementary material for: Papain Ameliorates Lipid Accumulation and Inflammation in High-Fat Diet-Induced Obesity Mice and 3T3-L1 Adipocytes via AMPK Activation
Source: Int J Mol Sci. 2021 Sep 14;22(18):9885. doi: 10.3390/ijms22189885 (PMC8468764; doi:10.3390/ijms22189885)
Supplement: Supplementary file 1 [file ijms-22-09885-s001.zip › ijms-1347364-supplementary.pdf]

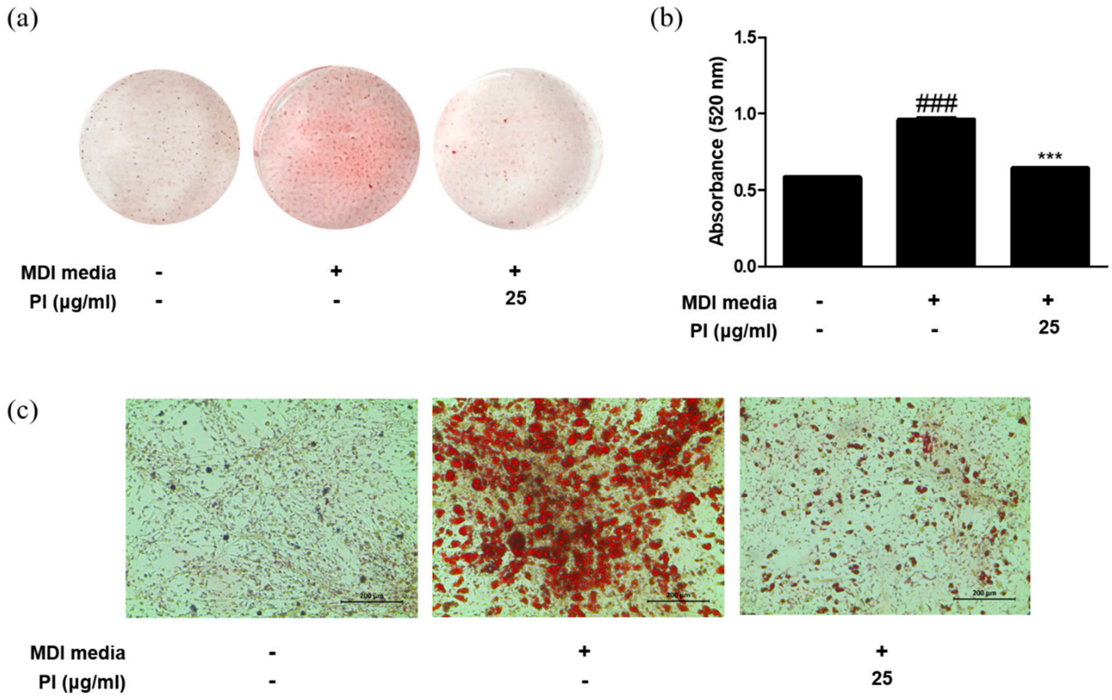

Supplementary Figure S1. Effects of Papain on adipocyte differentiation in MDI-treated rat primary adipocytes. (**a**) Oil red O staining 8 days after induction. (**b**) Quantitative analysis of Oil Red O staining. Oil Red O stained cells were extracted with isopropyl alcohol and the absorbance at 520 nm was measured. (**c**) Macroscopic shots of oil red O staining are shown. The values are given as the mean ± S.D. $^{###}p < 0.001$ vs. non-treated group; $^{***}p < 0.001$ vs. MDI-treated group
